# SEXUAL ORIENTATION AND AGING: A US–ENGLAND COMPARATIVE STUDY OF SAME-SEX AND DIFFERENT-SEX HOUSEHOLDS

**DOI:** 10.1093/geroni/igae098.0112

**Published:** 2024-12-31

**Authors:** Ariel Azar, Laia Becares, Dylan Kneale

**Affiliations:** Purdue University, West Lafayette, Indiana, United States; King’s College London, London, England, United Kingdom; University College London, london, England, United Kingdom

## Abstract

This study compares the health, income, and housing situation of same-sex and opposite-sex households in the United States and England. The research uses data from the English Longitudinal Study of Ageing (ELSA) and the U.S. Health and Retirement Study (HRS) to examine how these inequalities change over time. The study shows that individuals in same-sex households in both countries face distinct challenges in chronic health conditions, mental health status, and economic well-being. We argue that these challenges are due to the interaction between life transitions and varying policy, legislative, and societal attitudes toward same-sex relationships. For example, disparities in housing tenure and retirement income, which are more pronounced in the U.S. than in England, reflect these dynamic, divergent social and policy landscapes. The study takes into account relationship quality and satisfaction, gender, age, urban and rural living environments, and employment status. The findings highlight the cumulative impact of national contexts on the experiences of aging individuals in same-sex relationships. The study acknowledges data limitations due to the underrepresentation of sexual minorities in large nationally representative studies. However, the insights gained are essential for understanding how different policy contexts across the life course can shape the specific challenges and inequalities faced by this demographic group. The study emphasizes the need for informed policies and interventions.

